# Overestimation of the Subjective Experience of Time in Social Anxiety: Effects of Facial Expression, Gaze Direction, and Time Course

**DOI:** 10.3389/fpsyg.2016.00611

**Published:** 2016-04-29

**Authors:** Kenta Ishikawa, Matia Okubo

**Affiliations:** ^1^Center for Psychological Science, Institute for the Development of Social Intelligence, Senshu UniversityKawasaki, Japan; ^2^Department of Psychology, Senshu UniversityKawasaki, Japan

**Keywords:** social anxiety, time perception, facial expression, gaze direction

## Abstract

It is known that threatening stimuli increase emotional arousal, resulting in overestimating the subjective experience of passing time. Moreover, facial expressions and gaze direction interact to create socially threatening situations in people with social anxiety. The present study investigated the effect of social anxiety on the perceived duration of observing emotional faces with a direct or an averted gaze. Participants were divided into high, medium and low social anxiety groups based on social anxiety inventory scores. Participants then performed a temporal bisection task. Participants with high social anxiety provided larger overestimates for neutral faces with an averted gaze than those with low social anxiety in the second half of the task, whereas these differences were not found for angry face with direct and averted gaze. These results suggest that people with social anxiety perceive the duration of threatening situations as being longer than true durations based on objectively measured time.

## Introduction

According to the cognitive model of social anxiety (Clark and Wells, 1995; Rapee and Heimberg, 1997), people with social anxiety tend to have attentional biases directed toward potentially threatening stimuli such as negative facial expressions (Mogg et al., 2004), direct eye-contact (Horley et al., 2004), and social interactions with others (Amin et al., 1998; Stopa and Clark, 2000; Ishikawa et al., 2012). For example, Mogg et al. (2004) used a visual probe task and found that people with social anxiety had an attentional bias toward angry faces, as compared with happy and neutral faces. In addition to such attentional biases, socially anxious individuals tend to display a negative interpretational bias in social situations (Amin et al., 1998; Stopa and Clark, 2000; Ishikawa et al., 2012). Amin et al. (1998) found that socially anxious individuals interpreted social events more negatively than people with generalized anxiety disorder and normal controls, when reading about ambiguous social scenarios that can be interpreted in either a positive or negative light. A number of researchers have reported that these negative attentional and interpretational biases play an important role in the maintenance of social anxiety (Amin et al., 1998; Stopa and Clark, 2000; Horley et al., 2004; Mogg et al., 2004; Ishikawa et al., 2012).

Social anxiety-related negative biases may affect the subjective experience of time. It is well known that the subjective experience of time depends on both internal state and external situations (Droit-Volet et al., 2004; Tipples, 2008; Bar-Haim et al., 2010; Droit-Volet et al., 2010). For example, Bar-Haim et al. (2010) reported that people with generalized anxiety overestimated the duration for which fearful faces were presented, as compared to neutral faces. Such threat-related stimuli should increase emotional arousal and accelerate the speed of the internal clock, leading to an overestimation of perceived stimulus durations (Bar-Haim et al., 2010). Therefore, social anxiety-related negative biases might affect the subjective experience of time. Previous studies also suggested that this temporal overestimation for threatening stimuli may contribute to maintenance of anxiety and lead to unhelpful coping in people with anxiety disorders (Tipples, 2008).

Socially anxious individuals fear social evaluation, including rejection and angry displeasure (Clark and Wells, 1995; Rapee and Heimberg, 1997). As facial expression and gaze direction are important signals during social interactions and do indicate evaluation by others (Baron-Cohen, 1995; Adams and Kleck, 2003), people with social anxiety may well be afraid of these signals (Roelofs et al., 2010; Schmitz et al., 2012). Schmitz et al. (2012) found that people with high social anxiety exhibited an attentional bias toward neutral faces with averted gaze relative to low social anxiety individuals. As neutral faces with averted gaze can indicate rejection by others, an attentional bias toward such faces associated with social anxiety would appear to make sense (Schmitz et al., 2012). On the other hand, Roelofs et al. (2010) found that high social anxiety individuals tend to avoid looking at an angry face with a direct gaze, with no such tendency observed when the gaze was averted. An angry face with gaze focused directly on the other party represents a clear social threat, which may not be the case when the gaze is directed elsewhere. These results suggest that facial expressions and gaze direction interact to create socially threatening situations (Roelofs et al., 2010; Schmitz et al., 2012). In particular, we expect that both neutral faces with an averted gaze and angry faces with a direct gaze would be threatening to people with social anxiety and that such threatening stimuli should heighten emotional arousal, leading to overestimation of the perceived duration of observing these threatening stimuli. To our knowledge, no study has examined how people with social anxiety perceive threatening stimulus durations.

When investigating the effect of emotional arousal on perceived stimulus duration, the following points should be considered. First, the effect of anti-anxiety medication needs to be minimized during such investigations. It is well known that anti-anxiety medicine increases the subjective experience of time (Arushanyan et al., 2005) and works to decrease subjective arousals (Rammsayer, 1999). Therefore, an analog study employing participants not taking medication is needed to investigate the effect of emotional arousal on perceived stimulus duration.

Second, retroactive interference affects perceived stimulus duration, at least when a retrospective temporal task is used (e.g., a temporal bisection task with learned standard durations). One study reported that as trials proceeded, participants had difficulty remembering the standard durations of time that they had learned before target presentation (Bouton, 1993; Ogden et al., 2008). Ogden et al. (2008) demonstrated that temporal judgments became distorted after repeatedly observing stimulus patterns of various durations. According to Ogden et al. (2008), such distortion might be due to difficulty remembering standard durations. Socially anxious people negatively interpret social stimuli under difficult task conditions (Amin et al., 1998; Stopa and Clark, 2000; Ishikawa et al., 2012) Therefore, we hypothesized that difficulty remembering standard durations would produce a negative interpretation bias in temporal judgments, ultimately resulting in increased overestimation of perceived threatening stimulus durations. In addition, the effect of threatening stimuli on emotional arousal might be more pronounced after repeatedly observing such threatening stimuli. As the experiment proceeds, the speed of the internal clock should become more accelerated due to numerous presentations of threatening stimuli. To our knowledge, the effect of retroactive interference has not been taken into consideration in investigating the effect of emotional arousal on perceived duration of threatening stimuli. Therefore, experimental trials were divided into blocks, to examine the effect of retroactive interference on the perceived duration of threatening stimuli in social anxiety.

Based on these findings (Bouton, 1993; Droit-Volet et al., 2004, 2010; Ogden et al., 2008; Tipples, 2008; Bar-Haim et al., 2010; Roelofs et al., 2010; Schmitz et al., 2012), we hypothesized that people with social anxiety would overestimate the durations of socially threatening stimuli. Moreover, it was expected that facial expression and gaze direction would modulate this overestimation, which would become more exaggerated as the experiment proceeds, due to an emotional arousal-linked difficulty with remembering the standard stimulus duration. Our more specific hypotheses were as follows: (1) High social anxiety participants would overestimate the duration of both neutral and angry faces more than those with low social anxiety because participants with social anxiety are afraid of facial stimuli (Horley et al., 2004). (2) Participants with high social anxiety should overestimate the duration of neutral faces with an averted gaze more than those with low social anxiety, whereas no such difference would be found for neutral face durations with a direct gaze. (3) Participants with high social anxiety should overestimate durations for angry faces with a direct gaze more than those with low social anxiety, compared to angry faces with an averted gaze, because such stimuli can indicate interpersonal threat. (4) Duration overestimation of threatening stimuli in people with high social anxiety should become increasingly pronounced as the experiment proceeds. (5) Anxiety-related duration overestimation would not be observed in participants with low social anxiety.

## Materials and Methods

### Participants

Fifty-nine students (34 women, 25 men) at Senshu University took part in the present study. They were recruited at an introductory psychology class and received an extra course credit for the participation. Mean participant age was 21.1 years (*SD* = 2.8). Participants were not taking medication at the time of the present study. The Senshu University Human Research Ethics Committee approved this study (12-Dl107001-3). The experiment was carried out according to the Declaration of Helsinki. We obtained the written informed consent of the participants before commencing the study.

Participants were divided into three groups (high, medium, and low social anxiety) on the basis of scores on a Japanese version of the Brief Fear of Negative Evaluation scale (BFNE; Sasagawa et al., 2004). The BNFE includes 12 self-rated items that are each rated on a 5-point scale, ranging from 0 (“Not at all”) to 4 (“Extremely”). This scale has been widely used to discriminate socially anxious from non-anxious participants (Sasagawa et al., 2004). The BFNE has good internal consistency (Cronbach’s α = 0.92) and good test–retest reliability (*r* = 0.74; Sasagawa et al., 2004). Participants whose anxiety scores were in the highest 32.2% were included in the high social anxiety group (*n* = 19), and those in the lowest 33.9% were included in the low social anxiety group (*n* = 20). Other participants were included in the medium social anxiety group (*n* = 20).

### Materials

**FIGURE 1:**
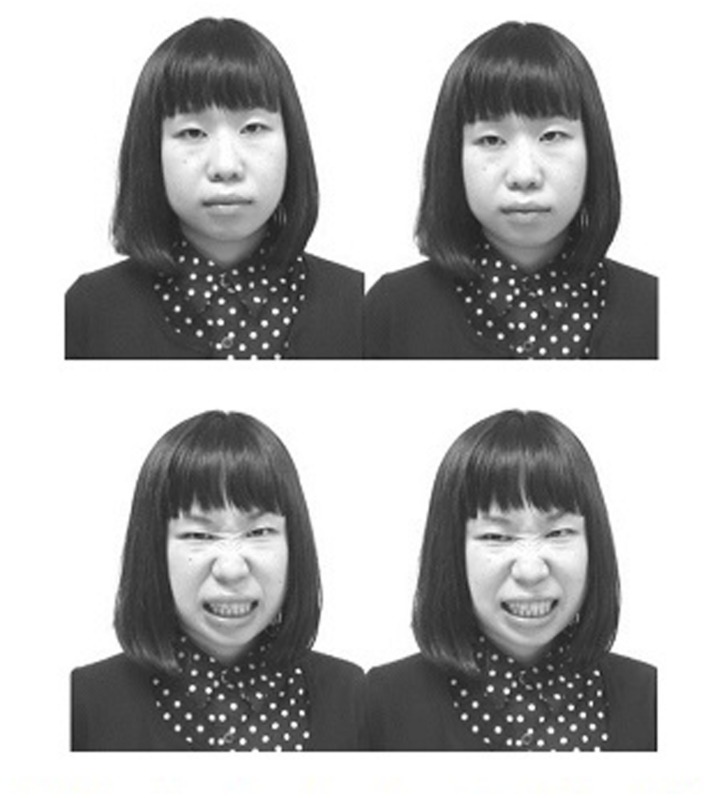
**Examples of face photographs used in the present study.** **(Upper left)** neuttal face with direct gaze, **(upper right)** neutral face with left averted gaze, **(under left)** angry face with direct gaze, **(under right)** angry face with left averted gaze.

We used 48 facial photographs defined by an orthogonal combination of eight Japanese models (four women and four men), two facial expressions (angry and neutral), and three gaze directions (direct, left, or right) (**Figure 1**). These photographs were used in our previous study (Ishikawa et al., 2012). To assess the validity of the emotional expressions depicted, 10 independent raters (six women and four men) classified the photographs according to six basic emotions (happy, surprise, anger, fear, sadness, and neutral). Raters’ classifications were accurate and highly consistent across facial expressions and gaze directions (angry with direct gaze = 96%, angry faces with averted gaze = 98%, neutral faces with direct gaze = 95%, neutral face with averted gaze = 91%). Each photograph subtended 14 cm in width and 17 cm in height, and appeared in gray scale on a 17-inch LCD monitor. We used a personal computer with a standard keyboard for controlling the experiment and for collecting the responses. The F and J keys of the keyboard were used to collect the responses.

### Procedure

We used a modified version of the temporal bisection task that was originally developed by Droit-Volet et al. (2004). This task has been used in previous studies to investigate the relationship between facial expressions and perceived stimulus durations (Droit-Volet et al., 2004; Tipples, 2008; Droit-Volet et al., 2010). Unlike in the original version, we manipulated facial expression and gaze direction. The experiment consisted of three phases (study, training and test). The study phase consisted of eight trials: participants were asked to learn two stimulus durations. Participants observed a neutral stimulus (a white oval) presented in the center of the computer screen, which was viewed for two standard durations: 400 ms (short) and 1600 ms (long). No response was required from the participants during the study phase. After the study phase, participants proceeded to the training phase. During the training phase that consisted of one block of 14 trials, the neutral stimulus (the white oval) was presented either for 400 or 1600 ms. Participants were asked to judge whether the stimulus was presented for the short or the long duration by pressing keys labeled short or long. The response keys were counterbalanced for each participant. At the end of the block, participants received feedback regarding the percentages of correct responses. When the percentages of correct responses were above 90%, participants proceeded to the test phase. If the percentages of correct responses were under 90%, participants repeated the study phase. During the test phase, the participants viewed facial photographs instead of the neutral stimulus. Facial expressions and gaze directions were manipulated in the photographs. Facial photographs were presented for one of seven durations (400, 600, 800, 1000, 1200, 1400, and 1600 ms). Participants judged whether the duration of a presentation was closer to the short or the long duration learned during the study phase. Participants were asked to respond as accurately as possible and were told that fast responses were not required. There were a total of 896 trials during the test phase. They consisted of 8 (model) × 2 (facial expression: angry and neutral) × 2 (gaze direction: direct and averted gaze) × 7 (durations: 400, 600, 800, 1000, 1200, 1400, and 1600 ms) = 224 distinct trials. The trials were repeated four times. The 896 trials were divided into two blocks (first and second block) of 448 trials. Each block consisted of two repetitions of 224 distinct trials, which were randomized across participants. Participants took a short break after every 112 trials and restarted the experiment at their own pace. Participants took approximately 1 h to finish the task. We used IBM SPSS Statistics version 20 for statistical analysis.

## Results

### Demographic Information of the High and Low Social Anxiety

**Table 1** shows demographic information for the high, medium and low social anxiety groups. It can be seen from **Table 1** that age [*F*(56) = 0.66, *p* = 0.51, $\eta_{p}^{2}$ = 0.02] and gender [χ^2^ (2) = 1.33, *p* = 0.51, Cramer’s *V* = 0.21] did not differ between the groups. BFNE score in participants with high social anxiety were significantly higher than the medium and low social anxiety, *F*(56) = 135.01, *p* < 0.001, $\eta_{p}^{2}$ = 0.82.

**Table 1:** Participant demographic information.

	High social anxiety (*n* = 19)			Medium social anxiety (*n* = 20)			Low social anxiety (*n* = 20)		
			
	Mean	*SD*s	95%	Cl	Mean	*SD*s	95%	CI	Mean	*SD*s	95% Cl
Age	20.42	1.46	[19.18, 21.65]	21.55	4.01	[20.34, 22.75]	20.96	1.73	[19.76, 22.16]
Gender (female %)	52.26			60			60		
BFNE	54.1	2.55	[51.79, 56.41]	45.65	3.93	[43.39, 47.90]	28.25	7.26	[25.99, 30.50]


*Means and standard deviations (*SD*s) are presented for each questionnaire. BFNE and *CI* stand for Brief Fear of Negative Evaluation Questionnaire and Confidence interval, respectively.*

### Temporal Bisection Task

The percentage of “long” responses was calculated for each experimental condition and subjected to a mixed-model Analysis of Variance (ANOVA), with social anxiety as a between-subject variable and facial expression, gaze direction, stimulus duration, and trial block as within-subject variables (**Tables 2** and **3**). We did not include the medium social anxiety group for ANOVA. The ANOVA revealed a significant main effect of Block, *F*(1,37) = 15.64, *p* < 0.001, $\eta_{p}^{2}$ = 0.29, indicating that the percentage of long responses was greater for the second block (*M* = 62.8% *SE* = 1.8) as compared to the first (*M* = 58.2% *SE* = 1.8). There was also a significant effect of stimulus duration, *F*(6,222) = 471.40, *p* < 0.001, $\eta_{p}^{2}$ = 0.92, showing that long responses increased with stimulus duration. The two-way interaction of social anxiety and Block was significant, *F*(1,37) = 9.78, *p <* 0.001, $\eta_{p}^{2}$ = 0.2, showing that the percentage of long responses in the second block (*M* = 66.2% *SE* = 2.7) was greater than in the first block (*M* = 58.3% *SE* = 2.6) among the high social anxiety participants. On the other hand, the percentage of long responses was not different between the first (*M* = 58.4% *SE* = 2.6) and second blocks (*M* = 59.3% *SE* = 2.6) for low social anxiety participants. There were no significant main effects of facial expression, *F*(1,37) = 1.75, *p* = 0.19, $\eta_{p}^{2}$ = 0.04, or gaze direction, *F*(1,37) = 0.89, *p* = 0.35, $\eta_{p}^{2}$ = 0.02. The three-way interaction was not significant, *F*(1,37) = 2.22, *p* = 0.14, $\eta_{p}^{2}$ = 0.05.

**Table 2:** Percentage of “long” responses for each experimental condition in the first block.

			High social anxiety			Low social anxiety		
				
Facial expression	Gaze direction	Duration	Mean	*SD*s	95% CI	Mean	*SD*s	95% CI
Angry	Direct gaze	400 ms	2.60	4.68	[-0.30, 5.60]	2.40	7.42	[–0.30, 5.30]
		600 ms	12.20	12.41	[3.10, 21.30]	12.20	24.29	[4.00, 21.70]
		800 ms	44.70	25.51	[31.70, 57.70]	37.50	29.56	[25.10, 50.50]
		1000 ms	72.40	24.78	[62.10, 82.60]	74.10	18.30	[65.30, 85.30]
		1200 ms	88.20	13.12	[82.00, 94.30]	89.90	12.89	[84.00, 96.00]
		1400 ms	96.70	5.87	[93.40, 100.00]	96.70	7.98	[93.30, 88.80]
		1600 ms	96.70	5.50	[94.50, 98.90]	98.20	3.57	[96.00, 100.30]
		
	Averted gaze	400 ms	2.30	5.05	[-1.00, 5.60]	3.30	8.48	[0.20, 6.60]
		600 ms	15.50	15.09	[6.50, 24.40]	12.80	22.15	[4.70, 22.10]
		800 ms	39.10	26.97	[26.40, 59.90]	37.20	27.09	[25.40, 50.20]
		1000 ms	73.00	25.57	[61.40, 84.60]	70.80	23.69	[60.60, 83.20]
		1200 ms	88.20	12.81	[82.20, 94.10]	90.50	12.58	[84.50, 96.10]
		1400 ms	94.70	5.46	[91.90, 97.50]	94.90	6.37	[92.60, 98.00]
		1600 ms	99.00	2.28	[97.60, 100.40]	98.50	3.44	[97.10, 99.80]

Neutral	Direct gaze	400 ms	1.30	3.26	[-1.80, 4.40]	3.00	8.72	[0.10, 6.10]
		600 ms	12.20	13.82	[2.80, 21.60]	13.70	24.57	[4.90, 23.20]
		800 ms	42.80	27.82	[29.70, 55.80]	36.90	27.73	[24.70, 50.30]
		1000 ms	71.40	24.11	[59.80, 83.00]	70.50	25.11	[59.90, 82.60]
		1200 ms	85.90	14.88	[80.00, 91.70]	88.10	9.09	[83.70, 95.00]
		1400 ms	94.70	5.83	[91.70, 97.80]	94.30	7.00	[91.40, 97.30]
		1600 ms	97.00	4.70	[94.50, 99.50]	97.30	5.88	[95.10, 99.90]
		
	Averted gaze	400 ms	2.60	5.10	[-0.70, 5.90]	3.00	8.48	[-0.10, 6.30]
		600 ms	12.80	17.14	[3.30, 22.40]	12.20	23.07	[3.50, 22.10]
		800 ms	40.80	24.11	[28.10, 53.50]	34.20	29.62	[22.60, 47.40]
		1000 ms	70.10	25.70	[58.20, 82.00]	70.80	24.87	[60.00, 83.20]
		1200 ms	86.50	12.21	[80.70, 92.30]	88.10	12.27	[83.10, 94.40]
		1400 ms	92.40	8.50	[88.10, 96.80]	94.60	9.78	[90.50, 98.90]
		1600 ms	97.40	3.69	[95.30, 99.50]	97.60	5.13	[95.40, 99.60]


**SD*s, standard deviation; *CI*, confidential interval.*

**Table 3:** Percentage of “long” responses for each experimental condition in the second block.

			High social anxiety			Low social anxiety		
				
Facial expression	Gaze direction	Duration	Mean	*SD*	95% CI	*Mean*	*SD*	95% Cl
Angry	Direct gaze	400 ms	5.30	7.10	[2.60, 7.90]	2.40	3.74	[-0.10, 5.10]
		600 ms	29.60	23.28	[20.30, 38.90]	11.30	15.56	[2.80, 21.00]
		800 ms	60.90	33.06	[46.10, 75.60]	43.80	29.52	[29.40, 58.10]
		1000 ms	80.60	25.72	[69.30, 91.90]	75.30	22.21	[64.00, 86.00]
		1200 ms	92.40	11.57	[86.90, 98.00]	89.90	12.04	[84.30, 95.10]
		1400 ms	96.00	7.35	[92.70, 99.20]	95.80	6.44	[92.50, 98.80]
		1600 ms	97.70	4.63	[94.60, 100.80]	96.10	8.18	[92.90, 99.00]
		
	Averted gaze	400 ms	7.60	8.97	[4.30, 10.90]	2.70	4.29	[-0.40, 6.00]
		600 ms	29.30	24.17	[20.20, 38.30]	11.00	12.43	[2.40, 20.10]
		800 ms	59.50	31.50	[45.20, 73.90]	44.90	29.26	[30.70, 58.60]
		1000 ms	77.50	23.46	[67.30, 87.70]	71.70	19.61	[61.90, 81.80]
		1200 ms	91.80	13.46	[86.40, 97.10]	94.30	8.72	[88.80, 99.20]
		1400 ms	96.70	5.12	[93.80, 99.70]	95.20	7.20	[92.10, 97.90]
		1600 ms	97.70	5.05	[95.10, 100.30]	97.00	5.91	[94.30, 99.40]

Neutral	Direct gaze	400 ms	6.20	7.59	[3.20, 9.30]	3.30	5.17	[0.10, 6.10]
		600 ms	29.30	23.21	[20.40, 38.20]	10.70	13.28	[2.30, 19.60]
		800 ms	62.50	33.38	[48.10, 76.90]	42.60	27.40	[28.50, 56.50]
		1000 ms	80.90	24.54	[70.40, 91.40]	79.50	19.90	[68.80, 89.30]
		1200 ms	89.50	15.84	[83.00, 95.90]	91.10	11.19	[84.60, 97.20]
		1400 ms	94.40	10.71	[90.30, 98.50]	95.80	6.12	[91.60, 99.60]
		1600 ms	97.40	4.21	[95.60, 99.10]	99.10	3.06	[97.40, 100.80]
		
	Averted gaze	400 ms	8.60	11.69	[4.50, 12.60]	1.50	3.44	[-2.40, 5.50]
		600 ms	31.60	24.20	[22.40, 40.70]	11.00	13.12	[2.00, 19.90]
		800 ms	60.90	32.31	[46.50, 75.20]	40.20	28.60	[26.70, 54.70]
		1000 ms	82.90	21.35	[72.80, 93.00]	70.20	21.54	[60.50, 80.20]
		1200 ms	92.10	11.80	[86.60, 97.60]	90.80	11.56	[85.30, 96.00]
		1400 ms	97.70	3.63	[94.80, 100.60]	96.70	7.98	[93.70, 99.40]
		1600 ms	96.40	6.19	[93.20, 99.50]	97.30	7.16	[94.10, 100.30]


**SD* and *CI* stand for standard deviation and confidential interval, respectively.*

Importantly, there was a four-way interaction between social anxiety, facial expression, gaze direction and block, *F*(1,37) = 4.98, *p* = 0.03, $\eta_{p}^{2}$ = 0.11. To clarify this interaction, we conducted a three-way repeated-measure ANOVA separately for each trial block. In the second block, the three-way interaction of social anxiety, facial expression and gaze direction was significant, *F*(1,37) = 6.79, *p* = 0.013, $\eta_{p}^{2}$ = 0.15. On the other hand, the three-way interaction was not significant in the first block, *F*(1,37) = 0.01, *p* = 0.98, $\eta_{p}^{2}$ > 0.01.

To further clarify the three-way interaction for the second block, we conducted a two-way repeated-measures ANOVA for each facial expression. The neutral expression condition produced a significant two-way interaction of social anxiety and gaze direction in the second block, *F*(1,37) = 10.21, *p* < 0.001, $\eta_{p}^{2}$ = 0.21, indicating that the percentage of long responses for averted gaze was greater in participants with high social anxiety than those with low social anxiety, whereas no such difference was found for direct gaze. Moreover, a main effect of social anxiety revealed that socially anxious participants tended to overestimate the duration of neutral faces relative to those with low social anxiety, *F* (1,37) = 3.52, *p* = 0.06, $\eta_{p}^{2}$ = 0.08. There was no significant effect of gaze direction, *F*(1,37) = 0.23, *p* = 0.63, $\eta_{p}^{2}$ > 0.01.

In the angry face condition, a main effect of social anxiety revealed that participants with high social anxiety tended to overestimate the duration of angry faces relative to those with low social anxiety. However, there was no significant main effect of social anxiety, *F*(1,37) = 3.05, *p* = 0.08, $\eta_{p}^{2}$ = 0.07. The two-way interaction of social anxiety and gaze direction [*F*(1,37) = 0.35, *p* = 0.55, $\eta_{p}^{2}$ > 0.01] and the main effect of gaze direction [*F*(1,37) = 0.01, *p* = 0.98, $\eta_{p}^{2}$ > 0.01) were not significant.

### Correlation between BFNE Scores and Increased Overestimation in the Second Block for Facial Expression and Gaze Direction

The four-way interaction described above suggests that overestimation of threatening stimulus durations was pronounced when socially anxious participants observed particular combinations of facial expression and gaze direction in the second block. To examine the relationship between social anxiety and this overestimation increase, we used the BFNE scores as a continuous variable (i.e., all participants, *N* = 59, were used) and conducted correlational analyses.

We defined the increased overestimation in the second block as the difference between the percentage of long responses in the first and second block for each condition (i.e., second block minus first block). Because participants accurately remembered the standard durations in the first block (as was indicated by high accuracy performance on the temporal bisection task), first block performance was used as a control condition and was compared with that in the second block. To examine such an increase, we conducted one-sample *t*-tests against zero for each condition. The overestimation increase for the second block was observed for angry faces with direct gaze [*M* = 3.56, *SE* = 1.02, *t*(58) = 3.49, *p* < 0.001, *d* = 0.64], angry faces with averted gaze [*M* = 3.58, *SE* = 0.99, *t*(58) = 3.62, *p* < 0.001, *d* = 0.66], neutral faces with direct gaze [*M* = 4.16, *SE* = 1.14, *t*(58) = 3.63, *p* < 0.001, *d* = 0.67] and neutral faces with averted gaze [*M* = 4.38, *SE* = 1.12, *t*(58) = 3.89, *p* < 0.001, *d* = 0.72). Moreover, we computed correlations between BFNE scores and the second block overestimation increase for each condition. BFNE scores were positively correlated with the increase for angry faces with direct (*r* = 0.35, *p* = 0.007) and averted gaze (*r* = 0.33, *p* = 0.011). BFNE scores were also positively correlated with the degree of emotional arousal for neutral faces with averted gaze (*r* = 0.40, *p* = 0.002). On the other hand, there was no correlation between BFNE scores and the increase in emotional arousal for neutral faces with directed gaze (*r* = 0.23, *p* = 0.09).

## Discussion

Participants with high social anxiety overestimated the perceived duration of threatening faces, in the second block of experimental trials. This result partially supported our first prediction that high social anxiety participants would overestimate the duration of threatening faces relative to those with low social anxiety. More specifically, high social anxiety participants overestimated the duration of neutral faces with an averted gaze in the second block. Furthermore, BFNE scores were positively correlated with duration overestimation for neutral faces with averted gaze in the second block, whereas no such correlation was observed for neutral faces with direct gaze. Previous studies have suggested that threatening stimuli increase perceived stimulus durations (Tipples, 2008; Bar-Haim et al., 2010). In our study, neutral faces with averted gaze that might indicate rejection by others (Schmitz et al., 2012) increased emotional arousal, and thereby accelerated the speed of the internal clock in participants with high social anxiety. In addition, these results were only found in the second block. This finding suggests that participants might have had difficulty in remembering the standard stimulus duration as the experiment proceeded, as has been demonstrated in previous studies (Bouton, 1993; Ogden et al., 2008). Indeed, many participants reported difficulty remembering the standard stimulus duration during a post-experiment interview. In addition, the repeated presentation of the threatening stimuli should increase negative interpretation bias, which might also lead to the overestimation observed here. The increases in arousal and interpretation bias should work in tandem to produce retrospective interference for high social anxiety participants, ultimately resulting in the overestimation of time observed here. Therefore, our results supported our hypothesis that the effect of social anxiety on emotional arousal would become more pronounced as the experiment progressed.

**FIGURE 2:**
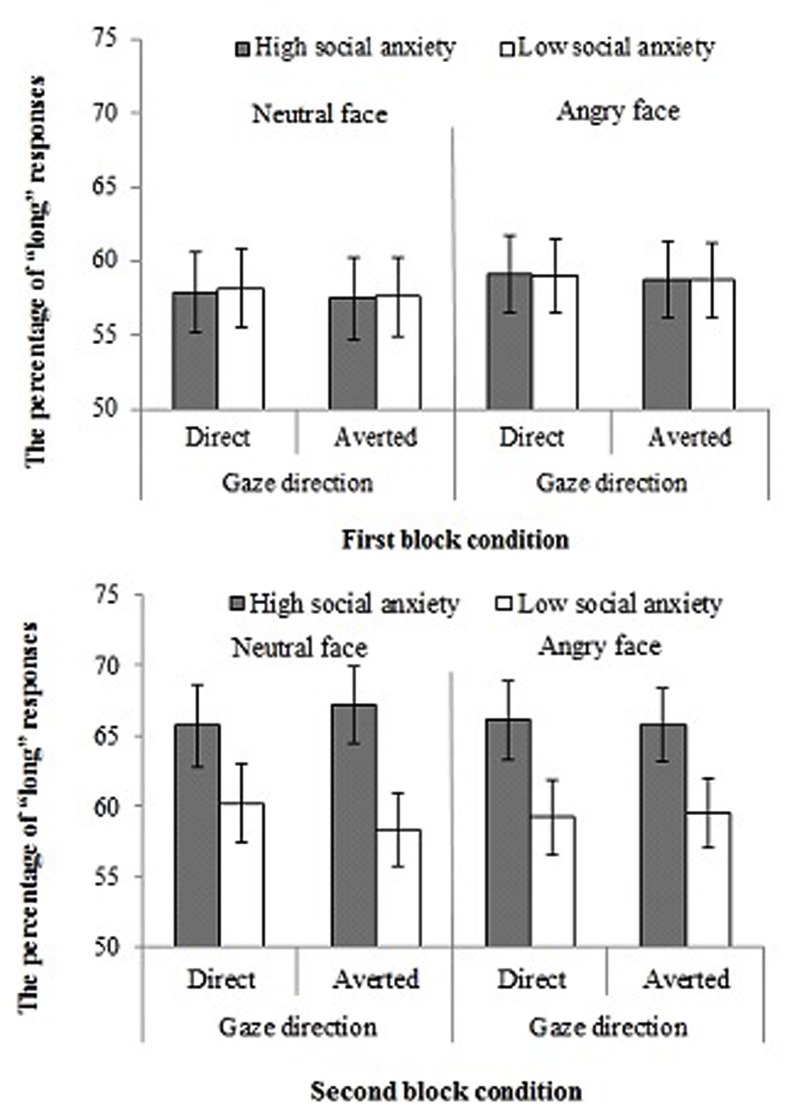
**Mean percentages of “long” responses during temporal bisection task.** Error bars indicate standard errors.

Our third prediction was partially supported. As can be seen in **Figure 2**, high social anxiety participants tended to overestimate angry face durations irrespective of gaze direction, again in the second block. BFNE scores were positively correlated with this overestimation. These results are partially consistent with previous studies reporting that people with social anxiety have a fear of angry faces with a direct gaze (Mogg et al., 2004; Roelofs et al., 2010) although the gaze direction did not modulate the size of overestimation in the present study. In addition, the size of overestimation was similar for angry faces and for neutral faces with an averted gaze. These results were somewhat unexpected. There would be two possible interpretations. First, emotional intensity for angry faces might be lower in the present study than in the previous ones (e.g., Droit-Volet et al., 2004, 2010; Tipples, 2008; Bar-Haim et al., 2010): the majority of the previous studies employed professional actors as models for photographs while we employed university students. In the present study angry faces may not be threatening enough even with a direct gaze. Second, neutral faces with an averted gaze may be as threating as angry faces with a direct gaze at least for people with high social anxiety (see Schmitz et al., 2012). The effect of the neutral face with averted gaze has not been tested thoroughly in the literature. Further research is needed to examine this issue.

Participants with low social anxiety overestimated the durations of neutral faces with a direct gaze compared to faces with an averted gaze, whereas this pattern was not observed for angry faces. These findings suggest that people with low social anxiety might have a tendency to show adaptive behaviors during social interactions. Facial expressions and gaze direction are important social cues that can signal evaluation by others (Baron-Cohen, 1995; Adams and Kleck, 2003). Therefore, people with low social anxiety might engage in a certain degree of avoidance of stimuli that signal the displeasure of others, such as neutral faces with gaze averted. Such a strategy might be used to mitigate social anxiety.

Many studies report gender differences in social anxiety (Turk et al., 1998; McLean et al., 2011; Caballo et al., 2014). However, we did not find a gender difference for BFNE scores here. Previous studies have reported that socially anxious men and women share similar levels of fear for social situations (Turk et al., 1998; McLean et al., 2011; Caballo et al., 2014). The fear of negative evaluation is one of the important characteristics of social anxiety (Clark and Wells, 1995; Rapee and Heimberg, 1997). Indeed, Turk et al. (1998) reported that fear of negative evaluation scores do not differ between men and women. This suggests that the effect of gender on social anxiety might be relatively small in the duration of the perceived threatening stimulus.

The present study has certain limitations. First, we did not directly measure emotional arousal during the task. In future research, it would be important to directly access emotional arousal by using direct measures such as skin conductance response and pupil dilation. Second, participants in the present study were not a clinical population. Therefore, our findings cannot be directly generalized to people with social anxiety disorders and the present results should be replicated with a clinical sample. Third, a main effect of block suggests that participants had difficulty remembering standard stimulus durations as the task proceeded. Therefore, it would be important to reduce the effects retroactive interference by shortening the amount of time in the experiment. It should be noted, however, that not only a main effect of block, but also an interaction of block and social anxiety was significant: the increase of overestimation was selectively observed for high social-anxiety participants. The increase of the difficulty in the second block may affect participants with high social anxiety more severely than those with low, producing the overestimation of stimulus duration for threatening stimuli. Fourth, we did not measure social desirability. Previous research has reported that anxiety is confounded with social desirability (Eysenck and Derakshan, 1997). Therefore, it would be needed to measure the social desirability in the further research.

The findings of this study suggest that people with social anxiety subjectively feel the duration of threatening situations to be longer than objective time. This overestimation of time could lead socially anxious people to process information in a biased fashion during engagements such as public speaking. In public speaking, socially anxious individuals might interpret neutral faces with averted gaze as a form of rejection. The resulting overestimation of time may lead to an avoidance tendency with regards to social situations, ultimately contributing to the development and maintenance of social anxiety disorder. Reducing such negative bias should be important for the treatment of people with social anxiety.

## Author Contributions

KI performed analysis on all samples, interpreted data, wrote the manuscript and acted as corresponding author. MO supervised development of the work, and helped in data interpretation and manuscript evaluation.

## Conflict of Interest Statement

The authors declare that the research was conducted in the absence of any commercial or financial relationships that could be construed as a potential conflict of interest.
